# The association of polypharmacy with COVID-19 outcomes independent of comorbidities in people with type 2 diabetes: implications for the unforeseen consequences of polypharmacy

**DOI:** 10.1097/XCE.0000000000000304

**Published:** 2024-05-23

**Authors:** Juhi K. Gupta, Rathi Ravindrarajah, George Tilston, Wiliam Ollier, Darren M. Ashcroft, Adrian H. Heald

**Affiliations:** a Division of Informatics, Imaging and Data Science, Faculty of Biology, Medicine and Health, University of Manchester; b Faculty of Science and Engineering, Manchester Metropolitan University; c Division of Pharmacy & Optometry, School of Health Sciences, Faculty of Biology, Medicine and Health, University of Manchester; d NIHR Greater Manchester Patient Safety Research Collaboration (PSRC), University of Manchester, Manchester; e Department of Diabetes and Endocrinology, Salford Royal NHS Foundation Trust, Salford and; f Faculty of Biology, Medicine and Health, University of Manchester, Manchester, UK

Previous studies of COVID-19 (SARS-CoV-2) infection in individuals with type 2 diabetes (T2D) have shown that the prescribing of certain medications influenced the likelihood of hospitalisation or death following COVID-19 infection [1–3] with COVID-19 vaccination mitigating that risk [4]. Many individuals in this population are often prescribed, and are taking, multiple medications as they live with other long-term conditions. We specifically wanted to focus on T2D individuals given the much greater risk of adverse consequences following a COVID-19 infection in this group.

To date, few studies have explored the associations between both polypharmacy and comorbidity with adverse health outcomes, such as hospital admission or death, post first infection of COVID-19 specifically in individuals with diabetes. In this retrospective cohort study, we aimed to investigate whether polypharmacy independent of comorbidity, conveyed a greater risk of adverse outcomes in people with diabetes after testing positive for COVID-19 infection.

Health outcome data were collected from 433 of 435 (99.5%) general practices in Greater Manchester. Data were de-identified at source and were extracted from the Great Manchester Care Record database. This was a retrospective cohort study with the period of follow-up 2020 to 2022 in relation to the main impact period of the COVID-19 pandemic. T2D individuals with a positive test for COVID-19 were included in this analysis.

Hospital admissions were recorded within 4 weeks after, or 2 weeks before a positive COVID-19 test (between January 2020 to May 2022). Across the Greater Manchester Region a total of 145 907 individuals were diagnosed to have T2D. The top six modal comorbidity groups identified in T2D were mental health conditions, hypertension, gastrointestinal/liver disease, pain, respiratory conditions/sinus-related and cardiovascular/cerebrovascular conditions. Of the individuals diagnosed with T2D, 69.9% were diagnosed with hypertension and 44.9% with mental health conditions.

Regarding individuals with T2D, an increased number of medications, from 11 to 15 was associated with increased risk of hospital admission following COVID-19 infection (*P* = 0.013; odds ratio (OR) [95% confidence interval (CI)] = 1.341 [1.063–1.692]) independent of the number of comorbidities, specific comorbidities and demographic factors, with a stronger association for >15 medications in regards to frequency of hospital admission: for 16–20 (*P* = 0.004; OR [95% CI] = 1.450 [1.124–1.870]); for >20 medications (*P* = 0.001; OR [95% CI] = 1.530 [1.191–1.966]).

In individuals with T2D, the number of deaths was 885 (0.6% of the total number of individuals diagnosed with T2DM). An increased number of medications, from 16 to 20 and >20 was associated with increased risk of death following COVID-19 infection (OR [95% CI] = 2.375 [1.306–4.319], *P* = 0.005; OR [95% CI] = 3.141 [1.755–5.621], *P* < 0.001 respectively) independent of the number of comorbidities, specific comorbidities and demographic factors.

In the multiple regression analysis a lower estimated glomerular filtration rate was associated with a higher likelihood of both hospital admission (OR [95% CI] = 1.431 (1.268–1.614), *P* < 0.001) and death following a COVID-19 infection (OR [95% CI] = 1.382 (1.078–1.771), *P* = 0.011). A higher BMI was associated with a higher likelihood of both hospital admission (OR [95% CI] = 1.011 (1.004–1.018), *P* < 0.001) and death (OR [95% CI] = 1.021 (1.007–1.036), *P* = 0.004) following a COVID-19 infection. Higher HbA1C was associated with a higher likelihood of hospital admission following COVID-19 infection (OR [95% CI] = 1.004 (1.001–1.006), *P* = 0.009), but not with death following a COVID-19 infection. There was no relation with cholesterol level regarding either hospital admission or death following a COVID-19 infection.

This study uniquely explored the relation between number of medications and adverse outcome from COVID-19 infection in individuals diagnosed with diabetes in the Greater Manchester population and is therefore distinct from our previous published studies in this area [2–4]. We found that the number of medications prescribed to an individual was associated with an increased risk of severe outcome from COVID-19 infection, in people living with T2D in relation both to death and hospital admission. Given the large proportion of individuals diagnosed with comorbidities, it was not unexpected that many of these individuals were prescribed multiple medications. However, the effect of multiple medications was independent of the presence of the most common comorbidities and demographic status.

It is certainly the case that people with for example difficult to treat hypertension and hyperglycaemia would be more likely to have more medication. However, we were here covering all prescriptions not just those related to cardiometabolic risk in relation to the possible interactions between medications from multiple drug classes.

Following on from our findings, after any serious viral infection in people with diabetes, it may be the case that those people with diabetes on the greatest number of medications are at an elevated risk of severe complications and of death. We suggest that every opportunity be taken safely to reduce medication load when people with diabetes have their routine reviews given the potential for unforeseen polydrug interactions.

We also identified that there were a large proportion of diagnoses of mental health conditions in people with T2D. The relation between physical health conditions and poor mental health is an area that is not fully understood as is the influence of polypharmacy [5].

It could be argued that polypharmacy is a marker of poor treatment adherence rather than being a causative factor per se in relation to an adverse outcome following a COVID-19 infection. We accept that this may be the case. However, this is not a question that we can answer with this dataset.

In conclusion, we have identified that the prescription of multiple medications in people living with diabetes is associated with a higher risk of adverse health outcome – hospitalisation and death, following acute COVID-19 infection.

This study has laid the foundation for future investigations into how complex pharmacotherapy may influence clinical outcome in unsuspected and unwanted ways. Understanding these relations can enhance risk stratification and evidence-based decision-making that may improve care and improve clinical outcomes for people with T2D following a COVID-19 or other viral infections.

## Acknowledgements

This work was supported by the Turing-Manchester Feasibility Project Funding.

R.R. performed the data cleaning and data analyses. J.K.G., W.O. and A.H.H. conceived the study. J.K.G. prepared the figures and the manuscript. G.T. extracted the original dataset. D.M.A., W.O. and A.H.H. contributed to all sections of the paper and D.M.A. provided expert input from a pharmacological perspective. All authors were involved in designing the study, interpreting results and the reviewing and editing of the manuscript.

This project was reviewed, and ethical approval for COVID-19 research was overseen by Health Innovation Manchester and granted by the Greater Manchester Care Record (GMCR) review board (ref: IDCR-RQ-046).

The datasets generated during and analysed during the current study are not publicly available as all data access is subject to review by Health Innovation Manchester.

### Conflicts of interest

There are no conflicts of interest.
